# Size-dependent redox behavior of iron observed by *in-situ* single nanoparticle spectro-microscopy on well-defined model systems

**DOI:** 10.1038/srep18818

**Published:** 2016-01-06

**Authors:** Waiz Karim, Armin Kleibert, Urs Hartfelder, Ana Balan, Jens Gobrecht, Jeroen A. van Bokhoven, Yasin Ekinci

**Affiliations:** 1 Laboratory for Micro and Nanotechnology, Paul Scherrer Institute, 5232 Villigen-PSI, Switzerland; 2 Institute for Chemical and Bioengineering, ETH Zurich, Switzerland; 3 Swiss Light Source, Paul Scherrer Institut, 5232 Villigen PSI, Switzerland; 4 Laboratory for Catalysis and Sustainable Chemistry, Paul Scherrer Institute, 5232 Villigen-PSI, Switzerland

## Abstract

Understanding the chemistry of nanoparticles is crucial in many applications. Their synthesis in a controlled manner and their characterization at the single particle level is essential to gain deeper insight into chemical mechanisms. In this work, single nanoparticle spectro-microscopy with top-down nanofabrication is demonstrated to study individual iron nanoparticles of nine different lateral dimensions from 80 nm down to 6 nm. The particles are probed simultaneously, under same conditions, during *in-situ* redox reaction using X-ray photoemission electron microscopy elucidating the size effect during the early stage of oxidation, yielding time-dependent evolution of iron oxides and the mechanism for the inter-conversion of oxides in nanoparticles. Fabrication of well-defined system followed by visualization and investigation of singled-out particles eliminates the ambiguities emerging from dispersed nanoparticles and reveals a significant increase in the initial rate of oxidation with decreasing size, but the reactivity per active site basis and the intrinsic chemical properties in the particles remain the same in the scale of interest. This advance of nanopatterning together with spatially-resolved single nanoparticle X-ray absorption spectroscopy will guide future discourse in understanding the impact of confinement of metal nanoparticles and pave way to solve fundamental questions in material science, chemical physics, magnetism, nanomedicine and nanocatalysis.

Iron is the fourth-most abundant element in the earth’s crust and exists in various oxidation states. The principal forms that occur naturally are ferrous Fe(II) and ferric Fe(III) iron. It is an integral part of a number of proteins and enzymes^1^ and the two oxidation states with the magnetic properties of iron oxides make it suitable for numerous biomedical applications and biochemical reactions^2^,^3^. Applications of iron and iron oxide nanoparticles have been explored in RF-mediated cell activation^4^, cancer immunotherapy^5^, medical imaging and drug delivery^6^, information storage^7^, wastewater treatment and ground water remediation^8^, among many others^9^. Iron is also an essential industrial catalyst in many reactions such as the Haber-Bosch process for ammonia synthesis^10^, Fischer–Tropsch synthesis^11^, and hydrogenation reactions^12^,^13^. It offers a low-cost and non-toxic alternative to rare and precious metal catalysts such as platinum. Research in the area of understanding iron and its oxides has a long history. Iron reacts with oxygen in water or moisture to form various insoluble iron oxides, described commonly as rust^14^, which limits applications of metallic iron. The interaction of oxygen with iron is important in a wide range of technological areas including corrosion, metallurgy, magnetism, catalysis and nanomedicine^4^,^6^,^11^,^15^,^16^. Understanding the size effects is of great interest and many studies have indicated that shape and size of nanoparticles considerably affect selectivity and activity of reactions involving metal catalysts but the impact of confinement of nanoparticles on the active sites is ambiguous^17^,^18^,^19^,^20^,^21^,^22^,^23^,^24^. The majority of studies in the literature concern the oxidation of iron sheets in ambient conditions and high temperature^15^,^16^. There is less work using oxygen and well-defined surfaces or iron nanoparticles and the size-dependent mechanism of the very early stages of oxidation has not been explained^25^,^26^,^27^. Bulk iron behaves differently from nanoparticles but very little knowledge exists at the nanoscale^28^,^29^,^30^ and, for a more thorough understanding, precise control and systematic characterization of such a nanoparticle system is needed. Studies of oxygen adsorption and oxide growth on iron have been reported using STEM (scanning transmission electron microscopy), LEED/AES (low-energy electron diffraction and Auger electron spectroscopy), and XPS (X-ray photoelectron spectroscopy), but none of these techniques allow visualization at the nanoscale of different sizes simultaneously and uncertainty over the kinetics, mechanism and structures evolving at the earliest stages of oxidation of iron remains^29^,^30^,^31^,^32^. Atomic-resolution high-angle annular dark-field (HAADF) imaging in aberration corrected STEM^33^,^34^, scanning transmission X-ray microscopy (STXM)^35^, high-resolution X-ray diffraction^36^ and ultra-fast 3D imaging^37^ can visualize catalyst materials but there is very limited possibility for surface-sensitive *in-situ* spectroscopy to directly observe chemical reactions on individual nanoparticles of various sizes together^38^. We leveraged the technique of X-ray photoemission electron microscopy (PEEM)^39^ which enabled high resolution imaging and simultaneous X-ray absorption spectroscopy (XAS) to elucidate the nanoparticle size effect.

Conventional techniques to synthesize iron nanoparticles, such as wet-chemical methods and sputter gas condensation either does not give well-defined feature sizes or lack precise positioning of the particles^19^,^40^,^41^,^42^. Most importantly, achieving more than one particle size with well-defined lateral order on the same support is generally not possible with any of these techniques. Combining the precision of top-down lithography^43^,^44^ to obtain single iron nanoparticles and the elemental sensitivity of the PEEM (Fig. 1a)^45^,^46^, we demonstrated single particle spectroscopy and performed *in-situ* studies simultaneously on a well-defined model systems consisting of ordered iron nanoparticles. We achieved the deposition of nanoparticles of controlled sizes and subsequently the synchronous spectroscopic characterization of individual nanoparticles of different sizes during reduction and oxidation. These developments open up new opportunities in design and synthesis of surfaces with controlled functionality and in applications of *in-situ* single particle spectro-microscopy for metallic nanocatalysis.

## Results

### Single particle spectro-microscopy on well-defined model system

**Figure 1:**
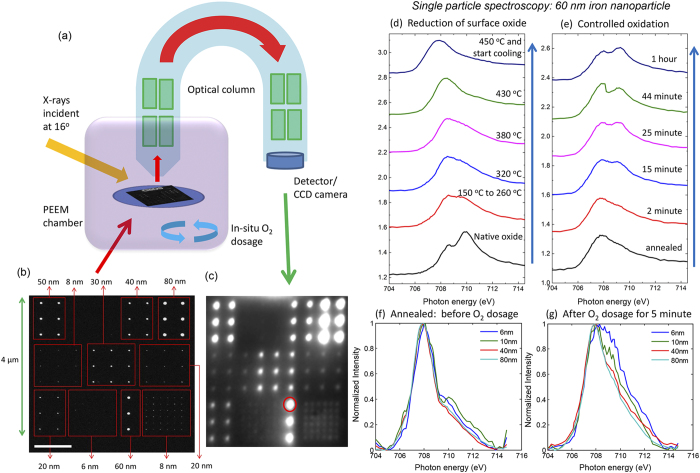
Single particle spectroscopy on iron nanoparticles. (**a**) Schematic of the PEEM setup for *in-situ* single nanoparticle spectro-microscopy. (**b**) SEM image of iron nanoparticles of nine different sizes (from 6 to 80 nm in diameter) in a 4 × 4 μm^2^ field-of-view fabricated using top-down nanofabrication with a height of 2.5 nm (scale bar 1 μm). (**c**) Elemental contrast image in the PEEM at Fe L_3_ edge where each bright spot corresponds to an iron nanoparticle. (**d**) XAS spectra at Fe L_3_ edge during different stages of annealing of a selected single 60 nm particle and obtaining pure iron by *in-situ* temperature-controlled reduction of surface oxide. (**e**) Evolution of XAS spectra of the 60 nm particle at different intervals of oxidation at 1 × 10^−8^ mbar. XAS spectra at Fe L_3_ edge of four different particle sizes (6, 10, 40, 80 nm) after (**f**) annealing at 450 **°**C where all the spectra are similar and resemble that of metallic iron, (**g**) after 5 minutes of oxygen dosage at 1 × 10^−8^ mbar showing largest extend of oxidation of the smaller nanoparticle.

Iron nanoparticles of nine different sizes, in the shape of pancake-like nanodots, were realized within a field-of-view of 4 × 4 μm^2^ on a silicon support with native silica using electron beam lithography (EBL) for exposure of PMMA resist employing proximity effect correction, followed by optimization of resist development, controlled thermal evaporation of pure iron, and lift-off (scanning electron microscope (SEM) image in Fig. 1b and see also Supplementary information S1). The size of the nanoparticles, i.e. the lateral diameter of the nanodots, achieved was between 6 nm and 80 nm with the thickness being 2.5 nm. In addition, iron alignment marker having a lateral size of 2.25 μm × 0.5 μm fabricated together with the nanoparticles served as the bulk reference during measurement. X-ray PEEM has a spatial resolution of about 50 to 100 nm, and to avoid interactions between the nanoparticles and overlapping of their signals, the inter-particle distance ranged from 250 nm to 500 nm. Since the PEEM is a surface-sensitive technique probing about 3 nm of the surface, the thickness of the particles was optimized to 2.5 nm so that the whole particle is probed by X-rays, which is an advantage over earlier studies performed at similar particle sizes.

The sample was introduced in the PEEM chamber, and linearly polarized X-rays at the synchrotron were incident at an oblique angle of 16 degrees. Secondary electrons emitted in response to the absorption of X-rays by the sample were recorded with a detector at the end of the optical column which converts the electron image to a photon image (Fig. 1c). This provides a spatial map of X-ray absorption cross-section and therefore the chemical fingerprint of the sample is recorded and visualized at different photon energies, which allows the selection of individual areas of interest on the sample and perform *in-situ* spectroscopy. Figure 1c shows the elemental contrast image recorded with the photon energy set to the absorption peak near the Fe L_3_-edge and each bright spot corresponds to the intensity of the nanoparticles at this photon energy (Supplementary information S2). This PEEM image, obtained by pixel-wise dividing the image near the Fe L_3_-edge and the pre-edge region, is a one-to-one equivalence of the same field-of-view in the SEM image of the model system in Fig. 1b and all the nanoparticles are probed simultaneously. To measure XAS spectra, PEEM images were recorded for a range of photon energies around the Fe L_3,2_-edges over the selected field of view of 4 × 4 μm^2^ resulting in the spectra of all sizes at the same experimental conditions in one measurement synchronously (Supplementary video). The series of images in each energy scan were corrected for drift, followed by selection of the desired nanoparticle to obtain the individual XAS spectra (Supplementary information S3). This resulting X-ray absorption signal from the single nanoparticle was compared for different sizes after normalizing each of these spectra with the XAS signal from an area without iron nanoparticles.

### *In-situ* reduction of surface oxide

Exposure of iron(0) to ambient environment after sample preparation leads to the formation of a thin oxide layer which increases in thickness upon oxidation at higher temperature^30^,^32^,^42^,^47^. The XAS spectra of the native oxide state, for a 60 nm particle in Fig. 1d, shows a resonance peak and a shoulder. To obtain metallic iron from the iron - iron oxide core-shell particles, we annealed the sample under vacuum at the base pressure of the PEEM which is better than 5 × 10^−10^ mbar. The reduction of surface oxide and simultaneous measurement of XAS, shown in Fig. 1d for the 60 nm particle at different temperatures, leads to metallic iron at 450 °C. The native amorphous silica surface of the substrate remains intact at this temperature. The chemical state of the nanoparticles in vacuum was unchanged till 150 °C above which the peak resonance starts to disappear. The shoulder peak gradually shifts as the temperature is increased and, at 450 °C, the metallic iron state was obtained with the single Fe L_3_ peak shifted by about 2 eV from the initial peak resonance of the native oxide. The elemental contrast image at the Fe L_3_-edge after annealing shows much higher and uniform intensity for all particles when compared to native oxide (Supplementary information S2). Once metallic iron was achieved, the sample was gradually cooled down to room temperature which induced no further change in the XAS spectra (Supplementary information S4). The XAS spectra of nanoparticles of all different sizes at this stage had similar spectra, as illustrated in Fig. 1f for four different sizes.

### Size effects of the re-oxidation process

Oxidation of metallic iron nanoparticles was carried out by dosing molecular oxygen at a partial pressure of 1 × 10^−8^ mbar for one hour. All the nine different sizes were simultaneously probed after various intervals at this pressure. Figure 1e shows XAS spectra illustrating the time-dependent evolution of oxide peaks during controlled *in-situ* oxidation of a particle of 60 nm diameter. As oxygen dosage was continued, the Fe L_3_ edge peak remains intact but there was an evolution of a strong peak at about 1.4 eV higher energy. The nanoparticles of all sizes were analyzed in the same manner and compared at each interval of oxygen dosage. Figure 1g compares four different particle sizes (6, 10, 40, 80 nm) after five minutes of oxygen dosage and a clear size-dependent behavior was observed at the early stage of oxidation. The smaller particles showed much faster oxidation compared to the larger ones, apparent from the degree of evolution of the oxide peak. This trend was observed during the complete oxidation process (Supplementary information S5).

### Mechanism of oxidation

We considered the detailed mechanism of oxidation to understand the observed effect of particle size. During oxidation, iron grows in layers consisting of FeO (ferrous oxide), Fe_3_O_4_ (ferrous ferric oxide) and Fe_2_O_3_ (ferric oxide)^16^,^27^,^30^,^32^,^42^,^48^,^49^. It is oxidized when exposed to air or oxygen regardless of the morphology or synthesis method. The phase composition during oxidation at higher temperature is well explained as a progression from Fe(0):FeO:Fe_3_O_4_:Fe_2_O_3_ but it is difficult to distinguish the phase and composition of the oxide layer as one moves from the inner iron(0) to the oxide interfaces during the early phase of oxidation at room temperature which takes place rapidly. At higher temperatures, it is known that distinct layers Fe, FeO, Fe_3_O_4_ and Fe_2_O_3_ are formed and for much longer duration of oxidation, extending for months, the iron core can be fully oxidized with a presence of a Kirkendall void in the centre of the particle^27^. We explain the growth of these oxides during the initial stages of oxidation and understood its dependence on the size of nanoparticles.

**Figure 2:**
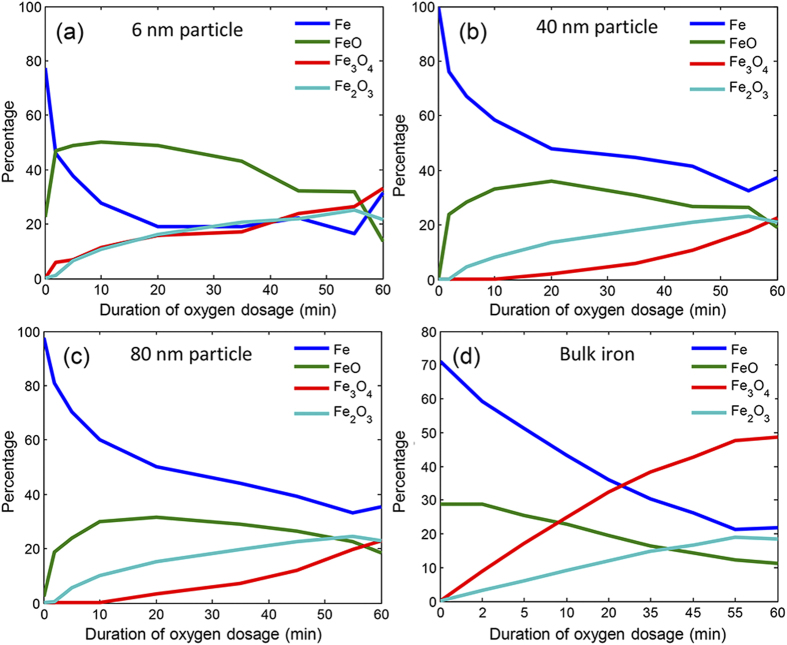
Change in concentration of Fe, FeO, Fe_2_O_3_, Fe_3_O_4_ during controlled oxygen dosage at 1 × 10^−8^ mbar over time observed for (a) iron particle with 6 nm diameter, (b) iron particle with 40 nm diameter, (c) iron particle with 80 nm diameter, and (d) bulk iron. Iron in bulk shows a different mechanism of inter-conversion of oxides compared to the nanoparticles. In addition, the rate of reduction of iron and inter-conversion of different oxides differ considerably as size of iron nanoparticles vary.

To elaborate on the composition of different oxides, fitting of reference spectra of FeO, Fe_3_O_4_ and Fe_2_O_3_ (Supplementary information S6) was performed on the spectra of single iron nanoparticles obtained at various intervals during the course of oxidation^48^. This provides the percentage concentration of the metallic iron and the oxides over the duration of oxygen dosage. The analysis was performed for all particles with size ranging from 6 nm to 80 nm and included the bulk surface, which were all probed simultaneously. Figure 2a–c shows the results for three selected particle sizes as well as for the bulk iron. For all sizes it was evident that the decrease in concentration of the metallic iron phase slows down after the early stage of rapid oxidation. The bulk iron exhibited a distinctly different rate of growth of the oxides as compared to any of the nanoparticles. This is in accordance with studies which claim that metallic nanoparticles behave differently compared to larger structures^18^,^20^,^21^,^22^,^23^,^24^,^28^,^29^,^30^,^50^.

The type of oxide formed is an important issue to be pursued since each oxide has distinct properties, affecting application in magnetism, optics, catalysis, wastewater treatment, and electronics. Fe_3_O_4_ and Fe_2_O_3_ are the most stable and common phases existing in nature and FeO is thermodynamically unstable^51^,^52^. The tendency of inter-conversion of oxides and non-stoichiometry arises due to different dispositions of ferrous and ferric ions in the lattice sites^53^. We observed that FeO forms as soon as oxidation begins. This phase is transient and rapidly converts into phases in which iron has higher oxidation state. There is a significant difference in the transition from FeO to Fe_3_O_4_ and/or Fe_2_O_3_ between the nanoparticles (Fig. 2a–c) and bulk iron (Fig. 2d). As oxidation of bulk iron begins, the concentration of metallic iron decreases and FeO formed on the surface further oxidizes to Fe_2_O_3_ and Fe_3_O_4_. Over the course of oxidation, there is a much stronger buildup of Fe_3_O_4_ and this continues to remain the dominant species (Fig. 2d).

For the nanoparticles, the phenomenon of oxidation is noticeably different (Fig. 2a–c). More precisely, during the initial stage of oxidation, FeO formed on the surface oxidizes almost completely to Fe_2_O_3_. As the concentration of metallic iron further reduces, Fe_2_O_3_ continues to increase with the evolution of Fe_3_O_4_ at a comparatively later stage. This occurs because the conversion of FeO to Fe_2_O_3_ is rapid and any intermediates, such as Fe_3_O_4_, in this process were only seen as traces. As the growth of Fe_2_O_3_ saturates after the initial phase of rapid oxidation, Fe_3_O_4_ increases in concentration with the decrease in FeO and metallic iron. For nanoparticles smaller than 10 nm in size, this transition from FeO to Fe_2_O_3_ is much faster; therefore Fe_2_O_3_ and Fe_3_O_4_ build up simultaneously (Fig. 2a). Additionally, a fit using the final most-oxidized state and initial metallic iron state showed there is a probability of more intermediates for the smallest particles (Supplementary information S7). Principal component analysis also revealed that as the size of the nanoparticle decreases, the number of species formed during oxidation increases (Supplementary information S8). The larger particles could be defined with lesser number of components as compared with the particles below 10 nm which also implies that other intermediate species may be present during early stages of oxidation.

**Figure 3:**
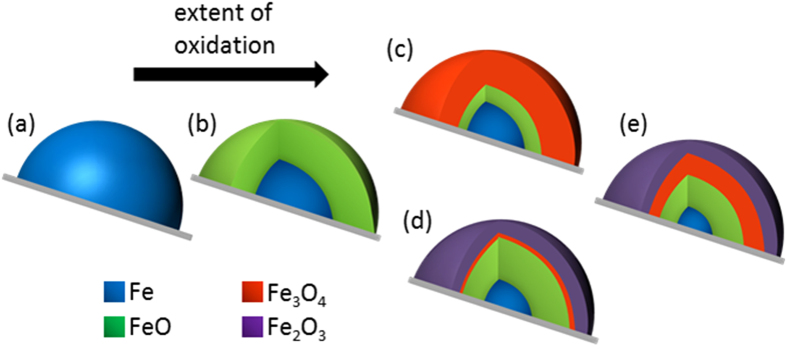
Mechanism of oxidation of iron nanoparticles. Schematic of the mechanism of oxidation for iron nanoparticles on silica support at ambient temperature: Early stage of oxidation of metallic iron (**a**) leads to formation of thermodynamically unstable FeO (**b**). Either directly from the intermediate Fe:FeO:Fe_3_O_4_ phase for bulk (**c**) or via a state with dominant Fe_2_O_3_ phase in nanoparticles (**d**), this is transformed to layers of Fe:FeO:Fe_3_O_4_:Fe_2_O_3_ (**e**).

The knowledge of this phase transformation during early stages of oxidation of iron nanoparticles unravels the mechanism of the formation of the oxide layers. Iron oxides are known to grow as multi-layered scales of FeO, Fe_3_O_4_ and Fe_2_O_3_ and that iron/iron oxide particles have core-shell structure. The mechanism of oxidation of the iron nanoparticles can be postulated using the schematic shown in Fig. 3. When oxygen is dosed at room temperature over metallic iron (Fig. 3a), the initial oxidation leads to the formation of FeO layer (Fig. 3b). The surface of this FeO layer transforms to the fully oxidized state of Fe_2_O_3_ for nanoparticles (Fig. 3d) due to rapid transformation of the intermediate Fe_3_O_4_ layer. This intermediate with surface Fe_3_O_4_ in Fig. 3c is short-lived. Upon further oxidation, the outermost Fe_2_O_3_ layer saturates at room temperature and the decrease in Fe and FeO concentration corresponds to further growth of the intermediate Fe_3_O_4_ layer. For the bulk surface, the state shown in Fig. 3d is not seen as the rate of transformation of the surface Fe_3_O_4_ to Fe_2_O_3_ is slow and therefore a much larger build up Fe_3_O_4_ was observed directly (Fig. 3e). As with nanoparticles after initial oxidation, upon further oxygen dosage, a decrease in the metallic core corresponds to increase in the FeO which now transforms to Fe_3_O_4_ phase in the region between the layers of FeO and Fe_2_O_3_ and small increase of Fe_2_O_3_ phase results from full oxidation of thin layers of Fe_3_O_4_ below the existing Fe_2_O_3_ layer. The size of the shell increases by two to three nanometers during the oxidation process at room temperature^32^,^41^.

## Discussion

**Figure 4:**
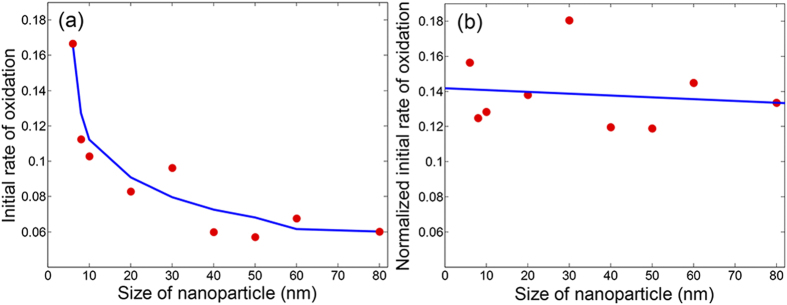
Particle size-effect during oxidation. (**a**) Initial rate of oxidation for all sizes of iron nanoparticles with a smoothened curve as an eye-guiding line. Particle sizes above 40 nm have constant rate of initial oxidation but this increases exponentially for smaller particles due to higher proportion of exposed surface area clearly showing a particle size-effect. (**b**) Initial rate of oxidation normalized for external surface area-to-volume ratio, which is the reactivity, of all particle sizes. The flat line is a best fit. Intrinsically in the atomic scale, the reactivity of particles at these dimensions and the behavior of active sites is still same.

To quantify the size-effect observed in the XAS spectra and phase transformation, the initial rates of oxidation were calculated for all particle sizes from the initial slope of the concentration of metallic iron in Fig. 2. Figure 4a shows this for nanoparticles of all the sizes which were probed simultaneously during the oxidation process. An exponential increase is seen with decreasing particle size. This is in accordance to the observation from the XAS spectra (Fig. 1g) where it was clear that the particles below 40 nm oxidized much faster. The nanoparticles with smaller radius of curvature have more structural disorder and less coincidence interfaces between crystallites, which enhance the diffusion of vacancies and iron cations^30^,^54^. For each single nanoparticle, the total exposed surface area is smaller as the size is reduced but this is accompanied by a higher surface-to-volume ratio (Supplementary information S9). This huge increase in exposed surface area in proportion to their volume influences the interaction of the nanoparticles with oxygen resulting in faster oxidation in smaller particles. This explains why in nano-sized particles, the build-up of Fe_3_O_4_ only occurs after a layer of Fe_2_O_3_ covers the surface, decreasing oxygen diffusion and slowing the oxidation process. The knowledge of the exact dimensions of the nanoparticles prepared using lithography allowed calculation of the initial rate of the reaction normalized by the surface-to-volume ratio of each nanoparticle (Fig. 4b). It represents averaged rate of the reaction at each active site and the intrinsic atomic properties of the nanoparticle. A flat line reveals that all the nanoparticles have the same atomic behavior. This is clear evidence that even though the faster oxidation is observed in smaller nanoparticles, this does not mean that that its intrinsic properties changed and neither does the reactivity per active site basis alters justifying that the mechanism of oxidation is same for all sizes that were probed. The atoms at the surface of any of these particles are equally reactive: with decreasing particle size, the relative number of active sites per unit area increases which increases the rate of oxidation. Nanoparticle size-effect in chemical reactions, that have been reported a number of times in the past, has been postulated as evidence to conclude difference in mechanism and propose unique properties of nanoparticles and its active sites based on size^17^,^22^,^28^,^30^. Our work confirms that this may not always be the case and the observed size effect is mainly because of increased surface-to-volume ratio but the overall property of the iron nanoparticles due to confinement at these dimensions does not change. This also implies that every size-effect seen in rate of reactions is not associated with change of intrinsic properties or increasing reactivity of catalytic metal nanoparticles but the opposite remains valid, i.e., if any special active sites are formed with further confinement at even smaller sizes, this will immediately impact in the reaction rate.

## Conclusion

We have achieved state-of-the-art top-down nanofabrication techniques for fabrication of well-defined model systems to simultaneously perform redox studies on iron nanoparticles of different sizes down to six nanometers. In conjunction with X-ray PEEM, we enabled *in-situ* single particle spectro-microscopy, visualized the reactions in the PEEM microscope and demonstrated the size-dependence of particle oxidation. This is an advance over previous studies on size effects of nanoparticles which have predominantly investigated each particle size as independent samples where the experimental setting between different sizes is prone to fluctuate at various stages of measurement. Here, at any instance of time, XAS spectra from each of the nine particle sizes could be extracted and compared with every other nanoparticle, all probed together under exactly the same environment, with complete control of size and order distinguishing individual iron nanoparticles with good spatial resolution. Monitoring the early stages of oxidation in controlled conditions reveals the mechanism of conversion of oxides to form the Fe:FeO:Fe_3_O_4_:Fe_2_O_3_ core-shell layers. The initial rate of particle oxidation depends on the number of exposed surface atoms but the high energy active sites for chemical reactions and the overall mechanism of oxidation remains the same with confinement at these particle sizes. Controlled nanopatterning to yield surfaces with particles of multiple sizes and the ability of single particle spectroscopy opens up new dimensions to solve fundamental questions in material science, nano-medicine, electronics, magnetism and catalysis. With further progress in the smallest achievable feature size and resolution using nanolithography, and improvement in space and time resolution of spectro-microscopy capabilities at PEEM, catalytic model systems with more complexities can be fabricated and studied in this manner which would bring a paradigm shift in understanding of nanoparticles and provide a fresh impetus to the field of nanocatalysis.

## Experimental Methods

### Top-down nanofabrication and characterization

Electron beam lithography (EBL) was used for fabrication of model systems consisting of iron nanoparticles of different sizes in the same field of view. Layout beamer was used to design the desired system (Supplementary information S1). PMMA resist is first spin-coated on a silicon wafer (with native oxide) and this is exposed using EBL. The area for each nanoparticle is patterned with proximity effect correction with exposure time optimized by fracture strategies to provide complete control over the shape and size. This results in patterning of hole arrays of desired dimensions. Controlled deposition of iron is done using the thermal evaporation technique. Subsequently, lift-off results in the desired nanoparticles ranging from 6 nm to 80 nm in size in the same field-of-view. For analysis during different stages of fabrication and to study the particle sizes, optical microscopy was done in conjunction with a scanning electron microscopy (Zeiss Supra VP55).

### Photoemission electron microscopy

Single-particle spectroscopy and microscopy were together carried out at the Surface/Interface:Microscopy (SIM) beamline^39^,^45^ at the Swiss Light Source using an Elmitec PEEM equipped with an energy analyzer. The beamline provides high brilliance X-ray light in the energy range of 130–2,000 eV. The image contrast in PEEM can arises from the element specificity, and chemical bonding in the sample. In order to obtain elemental contrast images, images at the iron L_3_ absorption edge and below the absorption edge were recorded sequentially and dividing the two images reduces topographic contrast and illumination inhomogeneities. The bright spots correspond to individual iron particles, and their variation in intensity is mostly related to the varying particle sizes and the chemical composition. The base pressure in the PEEM is 5 × 10^−10^ mbar and annealing of the samples was started at this condition. Dosage of oxygen in was controlled at a PEEM pressure of 1 × 10^−8^ mbar.

## Additional Information

**How to cite this article**: Karim, W. *et al.* Size-dependent redox behavior of iron observed by *in-situ* single nanoparticle spectro-microscopy on well-defined model systems. *Sci. Rep.* **6**, 18818; doi: 10.1038/srep18818 (2016).

## Supplementary Material

Supplementary Information

Supplementary Video
