# Supplementary material for: Size-dependent redox behavior of iron observed by in-situ single nanoparticle spectro-microscopy on well-defined model systems
Source: Sci Rep. 2016 Jan 6;6:18818. doi: 10.1038/srep18818 (PMC4702129; doi:10.1038/srep18818)
Supplement: Supplementary Information [file srep18818-s1.doc]

Supplementary Information

Size-dependent redox behavior of iron observed by *in-situ* single nanoparticle spectro-microscopy on well-defined model systems

**Waiz Karim^1, 2^, Armin Kleibert^3^, Urs Hartfelder^2,4^, Ana Balan^3^, Jens Gobrecht^1^, Jeroen A. van Bokhoven^2, 4*^ , and Yasin Ekinci^1*^**

^1^ Laboratory for Micro and Nanotechnology, Paul Scherrer Institute, 5232 Villigen-PSI, Switzerland,

^2^ Institute for Chemical and Bioengineering, ETH Zurich, Switzerland,

^3^ Swiss Light Source, Paul Scherrer Institut, 5232 Villigen PSI, Switzerland,

^4^ Laboratory for Catalysis and Sustainable Chemistry, Paul Scherrer Institute, 5232 Villigen-PSI, Switzerland.

Corresponding Authors

*Email: [jeroen.vanbokhoven@chem.ethz.ch](mailto:jeroen.vanbokhoven@chem.ethz.ch), *Email: [yasin.ekinci@psi.ch](mailto:yasin.ekinci@psi.ch)

The Supplementary information includes:

S1. Electron beam lithography layout and sample fabrication

S2. Elemental contrast images and single particle spectroscopy

S3. Spectra extraction for single particles

S4. Cooling of sample after annealing to metallic iron state

S5. Spectra evolution during oxygen dosage

S6. Reference spectra of different iron oxides

S7. Fitting the initial and final state of oxidation

S8. Principal component analysis

S9. Surface-to-volume ratio of the nanoparticles

S10. Supplementary Video.

S11. Supplementary Information references

Figures S1, S2, S3, S4, S5, S6, S7, and S8.

Table S1 and S2.

**S1. Electron Beam Lithography layout and sample fabrication**


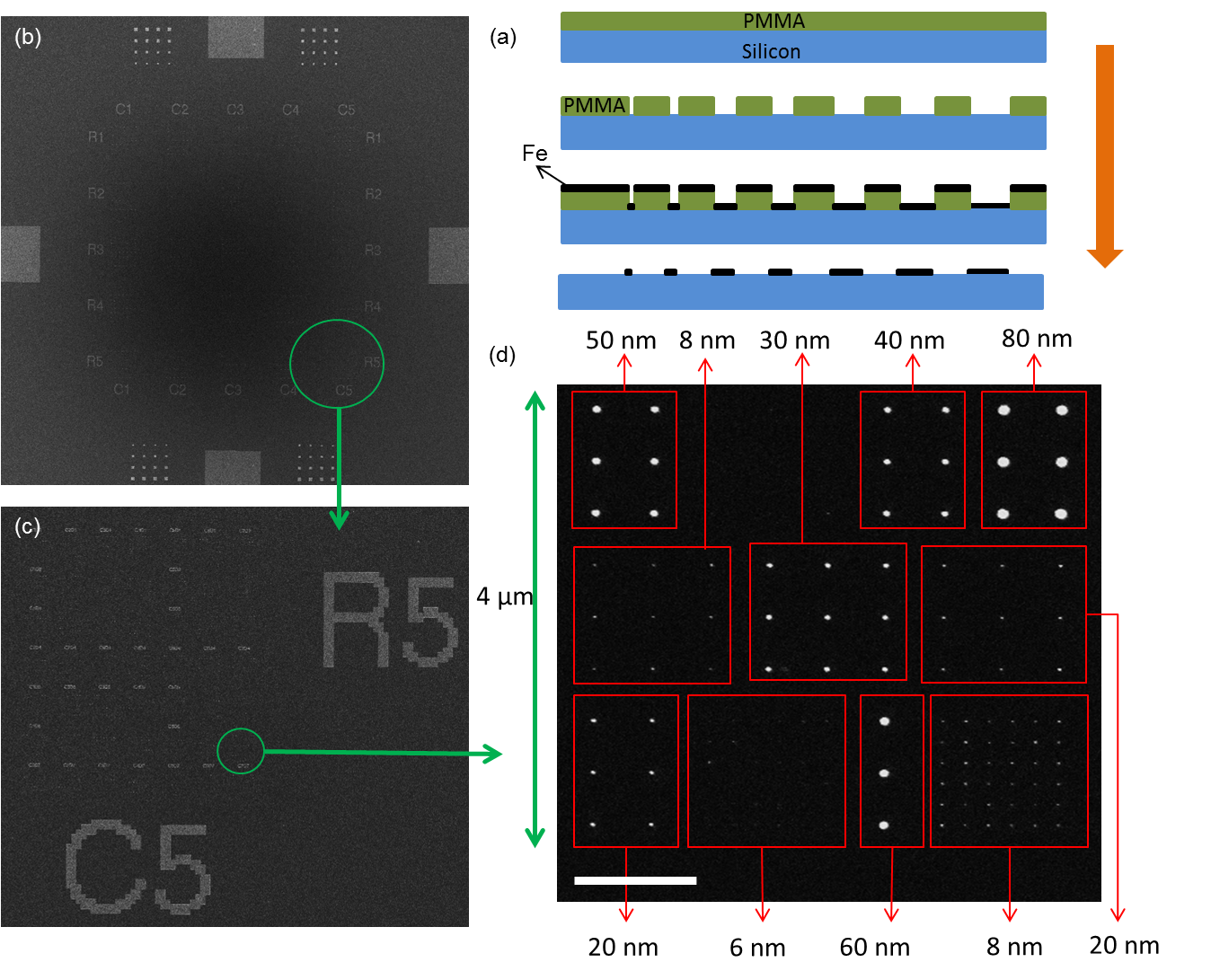


**Figure S1. Design and fabrication of model system for *in-situ* single particle spectro-microscopy in PEEM**. (a) Steps for fabrication involving spin-coating of a resist, electron beam lithography, thermal evaporation of iron and lift-off in acetone. To locate the exact area of study on a 1cm × 1cm silicon wafer inside the PEEM microscope, the e-beam layout was designed to incorporate iron markers exposed simultaneously along with the area with the nanoparticles. SEM image in (b) shows an area of 1 mm× 1mm in the center of the silicon substrate with native silica. A selected area from this field-of-view is shown at a higher magnification in (c) corresponding to about 1µm × 1µm area. The selected area inside this field-of-view corresponds to the model system with single iron nanoparticles of different sizes shown in the SEM image in (d). The same area was aligned for *in-situ* study under the PEEM microscope at the beamline. The scale bar here is 1 µm.

**S2. Elemental contrast images and single particle spectroscopy**

**
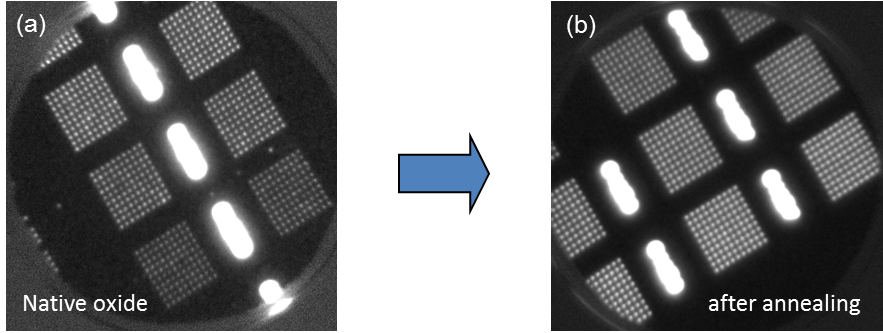
**

**Figure S2. Demonstration of change in elemental contrast images in the PEEM microscope at different conditions.** The elemental contrast image is obtained by dividing the image near the Fe L_3_-edge and the pre-edge region. Bright spots corresponds to the intensity the markers and the nanoparticles at this photon energy. PEEM elemental contrast images of a model system consisting of nanoparticles of only one size (60 nm particles) are shown. (a) shows the image with the native oxide and (b) shows the image after annealing in the PEEM vacuum. Since there is change in the chemical composition of the nanoparticles due to the reduction from native iron oxide to metallic iron, higher and uniform intensity is observed in (b) after the annealing.

**S3. Spectra extraction for single particles**


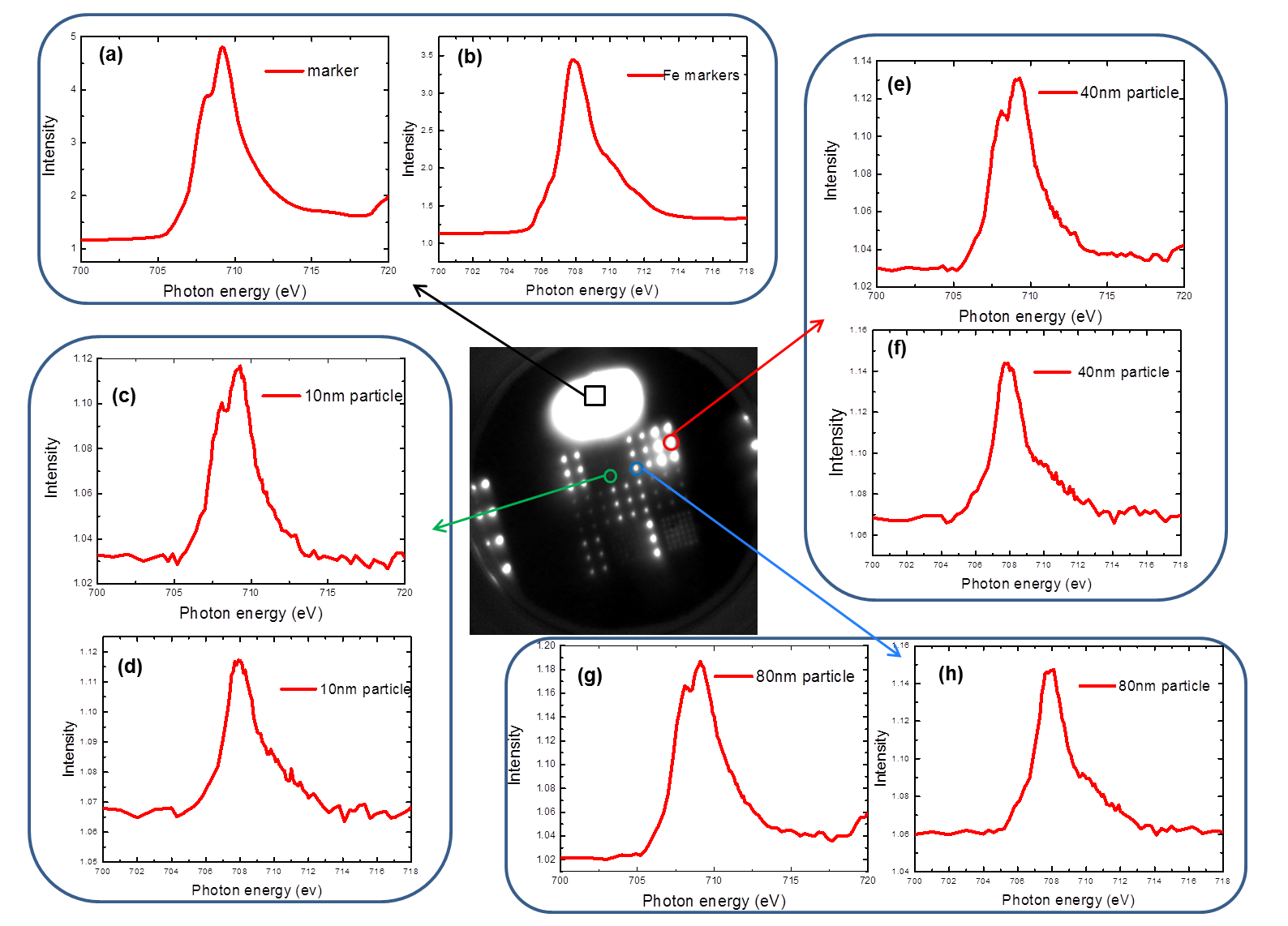


**Figure S3. *In-situ* single particle spectro-microscopy of iron nanoparticles**. Nine different sizes could be selected from the PEEM images and were probed simultaneously under the same condition at different stages of experiment. (a),(c),(e),(g) shows the XAS spectra of the native oxide and (b),(d),(f),(h) show the XAS spectra of metallic iron for the marker, 10 nm particle, 40 nm particle and 80 nm particle respectively as selected from the PEEM image.

**S4. Cooling of sample after annealing to metallic iron state**


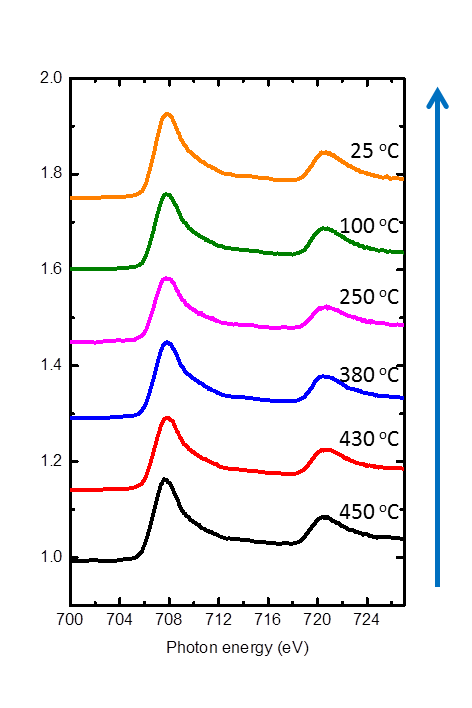


**Figure S4.** **Fe L_2,3_** **XAS spectra during intervals of cooling back to room temperature**. Metallic iron was obtained from the native oxide by annealing in vacuum to 450 °C as shown in Fig 1(d) for a 60 nm iron particle. No change in the XAS spectra of metallic iron nanoparticles is observed when cooling down the sample to room temperature in PEEM vacuum which is better than 5 × 10^-10^ mbar. Following this, *in-situ* oxidation was performed.

**S5. Spectra evolution during oxygen dosage**


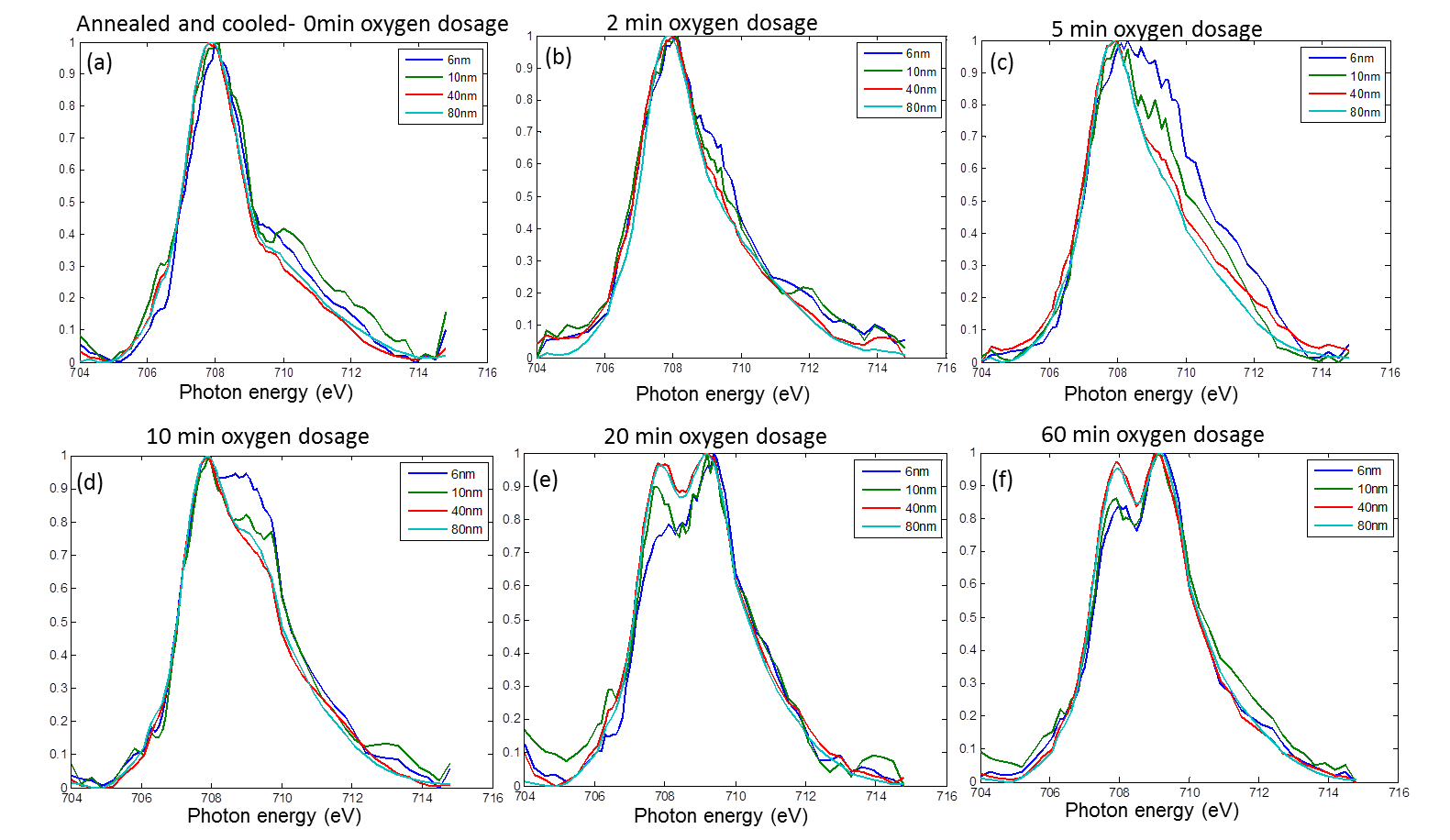


**Figure S5. Simultaneous comparison of XAS spectra from a 6 nm, 10 nm, 40 nm and 80 nm particle at different stages of oxidation at 1 × 10^-8^ mbar.** (a) The metallic iron stage after annealing in vacuum before beginning oxygen dosage; State at (b) 2 minutes of oxygen dosage, (c) 5 minutes of oxygen dosage, (d) 10 minutes of oxygen dosage, (e) 20 minutes of oxygen dosage, (f) 60 min minutes of oxygen dosage. As the size of nanoparticles decrease the evolution of oxide shoulder is much faster.

**S6. Reference spectra of different iron oxides**

**Figure S6. Reference spectra for Fe, FeO, Fe_3_O_4_ and Fe_2_O_3_ used for fitting in Figure 2.^1^**

**S7. Fitting the initial and final state of oxidation**


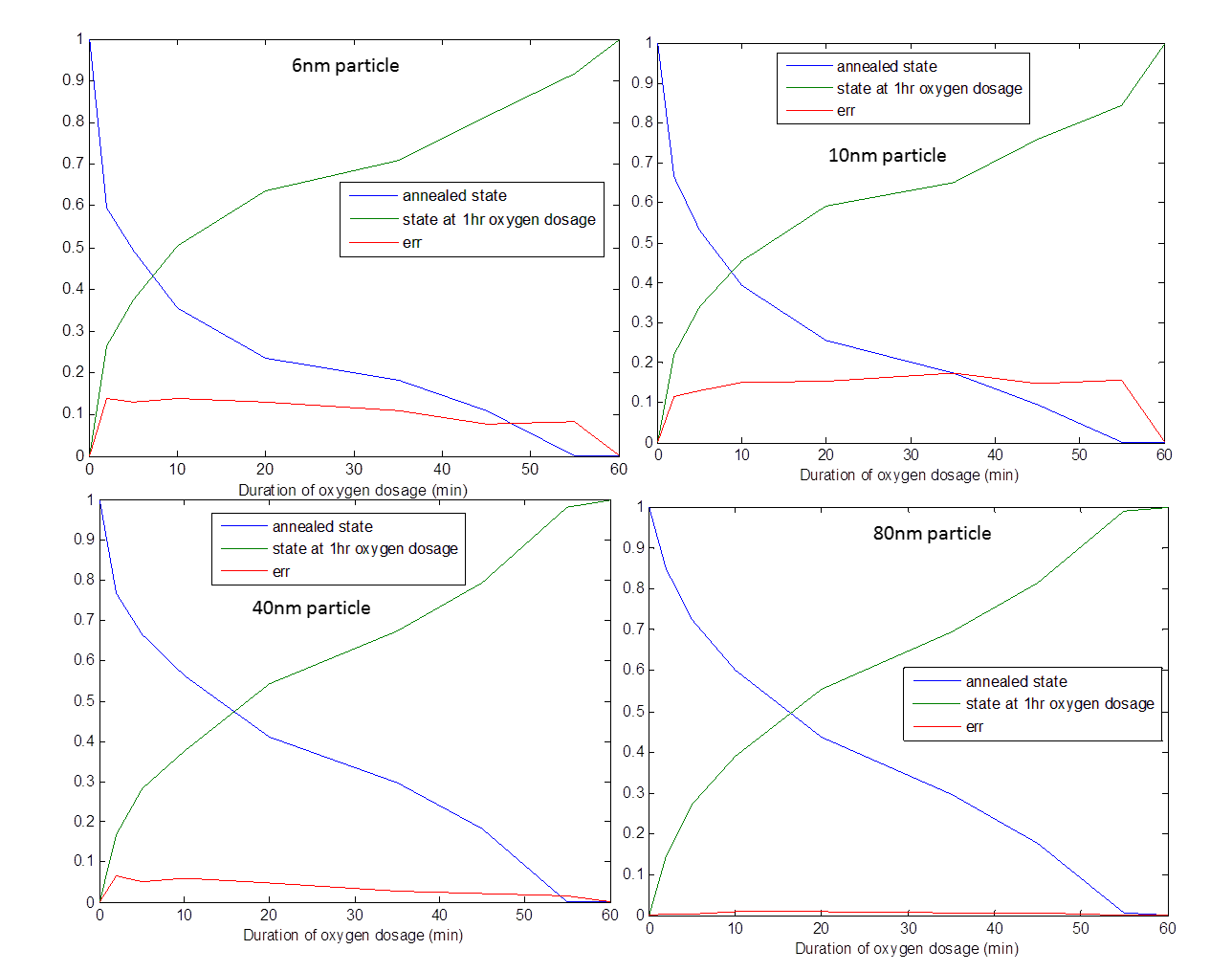


**Figure S7. Linear combination fits using initial (metallic iron state) and final state (after oxidation for one hour) for 6 nm, 10 nm, 40 nm and 80 nm iron particles with ‘err’ representing the residual in the fits.** The smaller the nanoparticle size, the higher the residual of the fit whch indicated a higher probability of more intermediates.

**S8. Principal component analysis**

Principal component analysis is widely used in data treatment to simplify complex data sets that contain multiple observations of many variables. It exploits the fact that some of these variables are correlated to simplify the data set, by transforming it in a way that replaces the original variables with principal components. This transformation is performed in a way that maximizes the variance in the data captured by the first few principle components, whereas the remaining components mainly capture the noise in the data set, and can therefore be omitted. Thus, a simplification of the dataset is achieved.

PCA has been successfully applied to XAS spectroscopy, since it permits investigating the number of species present in a spectrum or series of spectra.^2,3^ In order to analyze XAS spectra by PCA, it is necessary to interpolate all spectra to the same energy spacing and use consistent normalization. Then, the spectra can be concatenated into a single matrix (omitting the energy scale) and the PCA can be applied.

Table S1 shows the percentage of variance explained by the first 8 principal components for different particle size in the XAS data of the oxidation experiment. Figure S7 shows the cumulative variance explained for the same data set. These results show that for larger particles, a greater part of the changes can be explained by fewer principal components, in turn indicating fewer species with independent kinetic behavior (assuming equal noise level for all datasets).

**Table S**1 | The individual percentage variance of relevant principal components for iron nanoparticle of 6 nm, 10 nm, 40 nm and 80 nm size.

|  | **6nm dot** | **10nm dot** | **40nm dot** | **80nm dot** |
| --- | --- | --- | --- | --- |
| Principal  Component | Percentage  variance | Percentage  variance | Percentage  variance | Percentage  variance |
| 1  2  3  4  5  6  7  8 | 67.6971  20.0715  4.6993  3.1141  2.4066  0.9174  0.6686  0.4254 | 76.9631  6.4936  5.4746  3.6925  2.4387  2.1757  1.9028  0.8591 | 93.4884  3.7662  1.0402  0.6517  0.3871  0.2724  0.2098  0.1843 | 98.7823  0.8895  0.1202  0.0933  0.0561  0.0292  0.0155  0.0138 |

**Figure S8. Sum of the total percentage variance upto eight principal components for four different particle sizes during the course of oxidation.**  The total percentage in the figure represents the total combined percentage variance upto that principal component for nanoparticles of 6 nm, 10 nm 40 nm and 80 nm size.

**S9. Surface-to-volume ratio of the nanoparticles**

Sizes of the iron nanoparticles in Figure 1b represent the diameter ‘d’ of the nanoparticles. The diameter of nanoparticles probed simultaneously was 6 nm, 8 nm, 10 nm, 20 nm, 30 nm, 40 nm, 50 nm, 60 nm, and 80 nm. The height ‘h’ of each nanoparticle was constant at 2.5 nm so that the complete cylindrical particle was probed in the X-ray PEEM. The exposed surface area and exposed surface-to-volume ratio was calculated as follows:

Exposed surface area = (π × d^2^)/4 + (π × d × h)

Exposed surface to volume ratio = Exposed surface area/ ((π × d^2^ × h)/4)

Table S2 shows the values of the exposed surface to volume ratio for nanoparticle of all different sizes. Although the size of the nanoparticles decreases, the total exposed surface area in proportional to the volume of the nanoparticles increases. This leads to an increase in the rate of early stages of oxidation for smaller sizes as seen in Figure 4a. The rate of reaction normalized for the exposed surface area to volume ratio reveals that there is no change in these sizes and the intrinsic properties of the active sites remain the same at these dimensions as seen in Figure 4b.

**Table S**2 | Exposed surface area and surface-to-volume ratio of single nanoparticle from 6 nm to 80 nm in size, all probed simultaneously in the PEEM under same conditions.

| **Size (nm)** | **6** | **8** | **10** | **20** | **30** | **40** | **50** | **60** | **80** |
| --- | --- | --- | --- | --- | --- | --- | --- | --- | --- |
| **Exposed surface area (nm^2^)** | 75.40 | 113.10 | 157.08 | 471.24 | 942.48 | 1570.80 | 2356.19 | 3298.67 | 5654.87 |
| **Exposed surface-to-volume ratio (nm^-1^)** | 1.07 | 0.9 | 0.8 | 0.6 | 0.53 | 0.5 | 0.48 | 0.47 | 0.45 |

**S10. Supplementary Video**: Visualization of x-ray abosorption spectra of individual iron nanoparticles:

Simultaneously microscopy and spectroscopy of iron nanoparticles of nine different sizes is shown in the movie. It combined PEEM images for the range of photon energies around the Fe L_3,2_-edges over the selected field of view. The intensity varies corresponding to the intensity of the nanoparticles at the photon energy. This very weak in the pre-edge and post-edge region, has a peak intensity at the Fe L_3,2_-edges. Individual nanoparticles are then be selected to extract XAS spectra from a single nanoparticle.

**S11. Supplementary Information references**:

1 Regan, T. J. *et al.* Chemical effects at metal/oxide interfaces studied by x-ray-absorption spectroscopy. *Physical Review B* **64**, 214422, (2001).

2 Wasserman, S. The analysis of mixtures: application of principal component analysis to XAS spectra. *Le Journal de Physique IV* **7**, C2-203-C202-205, (1997).

3 Fay, M. J., Proctor, A., Hoffmann, D. P., Houalla, M. & Hercules, D. M. Determination of the Mo surface environment of Mo/TiO2 catalysts by EXAFS, XANES and PCA. *Microchimica Acta* **109**, 281-293, (1992).
